# Measurable Genomic Changes in Mycobacterium avium subsp. *hominissuis* after Long-Term Adaptation in Acanthamoeba lenticulata and Reduced Persistence in Macrophages

**DOI:** 10.1128/JB.00257-20

**Published:** 2021-02-22

**Authors:** Nabeeh A. Hasan, Grant J. Norton, Ravleen Virdi, L. Elaine Epperson, Charmie K. Vang, Brandon Hellbusch, Xiyuan Bai, Edward D. Chan, Michael Strong, Jennifer R. Honda

**Affiliations:** a Center for Genes, Environment and Health, National Jewish Health, Denver, Colorado, USA; b Advanced Diagnostic Laboratories, Complement Lab, National Jewish Health, Denver, Colorado, USA; c Department of Medicine and Academic Affairs, National Jewish Health, Denver, Colorado, USA; d Division of Pulmonary Science and Critical Care Medicine, University of Colorado Denver, Anschutz Medical Campus, Aurora, Colorado, USA; e Department of Medicine, Rocky Mountain Regional Veterans Affairs Medical Center, Denver, Colorado, USA; Brigham and Women's Hospital/Harvard Medical School

**Keywords:** *Mycobacterium avium* subspecies *hominissuis*, *Acanthamoeba lenticulata*, whole-genome sequencing, persistence, human THP-1 macrophages

## Abstract

Free-living amoebae are ubiquitous in aquatic environments and act as environmental reservoirs for nontuberculous mycobacteria. Mycobacterium avium subsp. *hominissuis* recovered from *Acanthamoeba* has been demonstrated to be more virulent in both human and murine models. Here, we investigate the persistence of M. avium subsp. *hominissuis* after short-term (2 weeks) and long-term (42 weeks) coculture in Acanthamoeba lenticulata. We hypothesize that *A. lenticulata-*adapted M. avium subsp. *hominissuis* demonstrates phenotypic and genomic changes facilitating intracellular persistence in naive *Acanthamoeba* and human macrophages. M. avium subsp. *hominissuis* CFU in coculture with *A. lenticulata* were recorded every 2 weeks for up to 60 weeks. While *A. lenticulata*-associated M. avium subsp. *hominissuis* CFU did not significantly change across 60 weeks of coculture, longer adaptation time in amoebae reduced the colony size. Isolates recovered after 2 or 42 weeks of amoeba coculture were referred to as “early-adapted” and “late-adapted” M. avium subsp. *hominissuis*, respectively. Whole-genome sequencing was performed on amoeba-adapted isolates with pan-genome comparisons to the original M. avium subsp. *hominissuis* isolate. Next, amoeba-adapted isolates were assessed for their persistence in *A. lenticulata*, Acanthamoeba castellanii, and human THP-1 macrophages. Multiplex cytokine/chemokine analyses were conducted on THP-1 culture supernatants. Compared to the original isolate, counts of late-adapted M. avium subsp. *hominissuis* were reduced in *Acanthamoeba* and, contrary to expectations, lower counts were also observed in THP-1 macrophages, with concomitant decreases in tumor necrosis factor alpha (TNF-α), interleukin 6 (IL-6), and macrophage inflammatory protein 1 beta (MIP-1β), suggesting that host adaptation may influence the inflammatory properties of M. avium.

**IMPORTANCE** Short-term interaction between *Acanthamoeba* and M. avium has been demonstrated to increase infectivity in human and murine models of infection, establishing the paradigm that amoebae “train” M. avium in the environment by selecting for phenotypes capable of enduring in human cells. We investigated this phenomenon further by determining the consequence of long-term amoeba adaptation on M. avium subsp. *hominissuis* persistence in host cells. We monitored genomic changes across long-term *Acanthamoeba* coculture and report significant changes to the M. avium subsp. *hominissuis* genome in response to amoeba adaptation and reduced colony size. Furthermore, we examined isolates cocultured with *A. lenticulata* for 2 or 42 weeks and provide biological evidence that long-term coculture in amoebae reduces M. avium persistence in human macrophages.

## Abstract (precis)

Short-term interaction between *Acanthamoeba* and M. avium has been demonstrated to increase infectivity in human and murine models of infection, establishing the paradigm that amoebae “train” M. avium in the environment by selecting for phenotypes capable of enduring in human cells. We investigated this phenomenon further by determining the consequence of long-term amoebae adaptation on M. avium subsp. *hominissuis* persistence in host cells.

## INTRODUCTION

Nontuberculous mycobacteria (NTM) are of growing concern globally because of their abundance in the environment (1) and their ability to cause chronic opportunistic pulmonary disease. Mycobacterium avium subsp. *hominissuis* and other members of the M. avium complex (MAC; i.e., Mycobacterium chimaera and Mycobacterium intracellulare) are predominantly responsible for clinical respiratory disease (2). M. avium subsp. *hominissuis* is a recognized endosymbiont of unicellular free-living amoebae (FLA), which coexist with NTM in freshwater systems and soil, providing protection from water disinfection processes and antimicrobial agents (3–8). In their trophozoite form, FLA act as environmental phagocytes that engulf debris and bacteria. However, a number of amoeba-resistant mycobacteria, including M. avium subsp. *hominissuis*, avoid intracellular phagolysosome-mediated killing by FLA, which facilitates their intracellular survival and persistence in the environment (9, 10).

Previous work indicates that FLA function as Trojan horses for dispersal of (myco)bacteria in the environment (11, 12), as well as training fields or biological gyms by exerting selective pressure on endosymbionts toward phenotypes that facilitate survival in human macrophages and evasion of host immune responses (13, 14). After several days of intracellular growth in *Acanthamoeba*, M. avium subsp. *hominissuis* shows increased entry and replication in both human monocyte‐derived macrophages and epithelial cells, as well as enhanced virulence in C57BL/6 bg^+^/bg^+^ mice (15). FLA may also serve as a cellular hub where genetic material can be exchanged among their bacterial endosymbionts (16).

While previous studies have focused on the interactions between NTM and amoebae after acute infection, e.g., 1 h, 3 days (15, 17), or 4 days postinfection (18), none have examined how M. avium subsp. *hominissuis* persistence changes after intermediate (2 weeks) and long-term (42 weeks) coculture in *Acanthamoeba*. While prior reports have suggested selection for more virulent M. avium subsp. *hominissuis* phenotypes after short-term coculture with Acanthamoeba castellanii (15), the impacts of longer periods of coculture on the persistence of NTM have not been explored. Here, we used bacterial whole-genome sequencing (WGS) to investigate M. avium subsp. *hominissuis* genomic changes associated with long-term *Acanthamoeba lenticulata* adaptation. We also demonstrate that extended adaptation time in *A. lenticulata* significantly decreases the ability of M. avium subsp. *hominissuis* to survive both in naive *Acanthamoeba* and human macrophages, with reduced persistence and reduced capacity to elicit host-protective immune responses.

(A portion of this work was published as a conference abstract [19].)

## RESULTS

### M. avium subsp. *hominissuis* burden remains stable across extended coculture in *A. lenticulata*.

**FIG 1:**
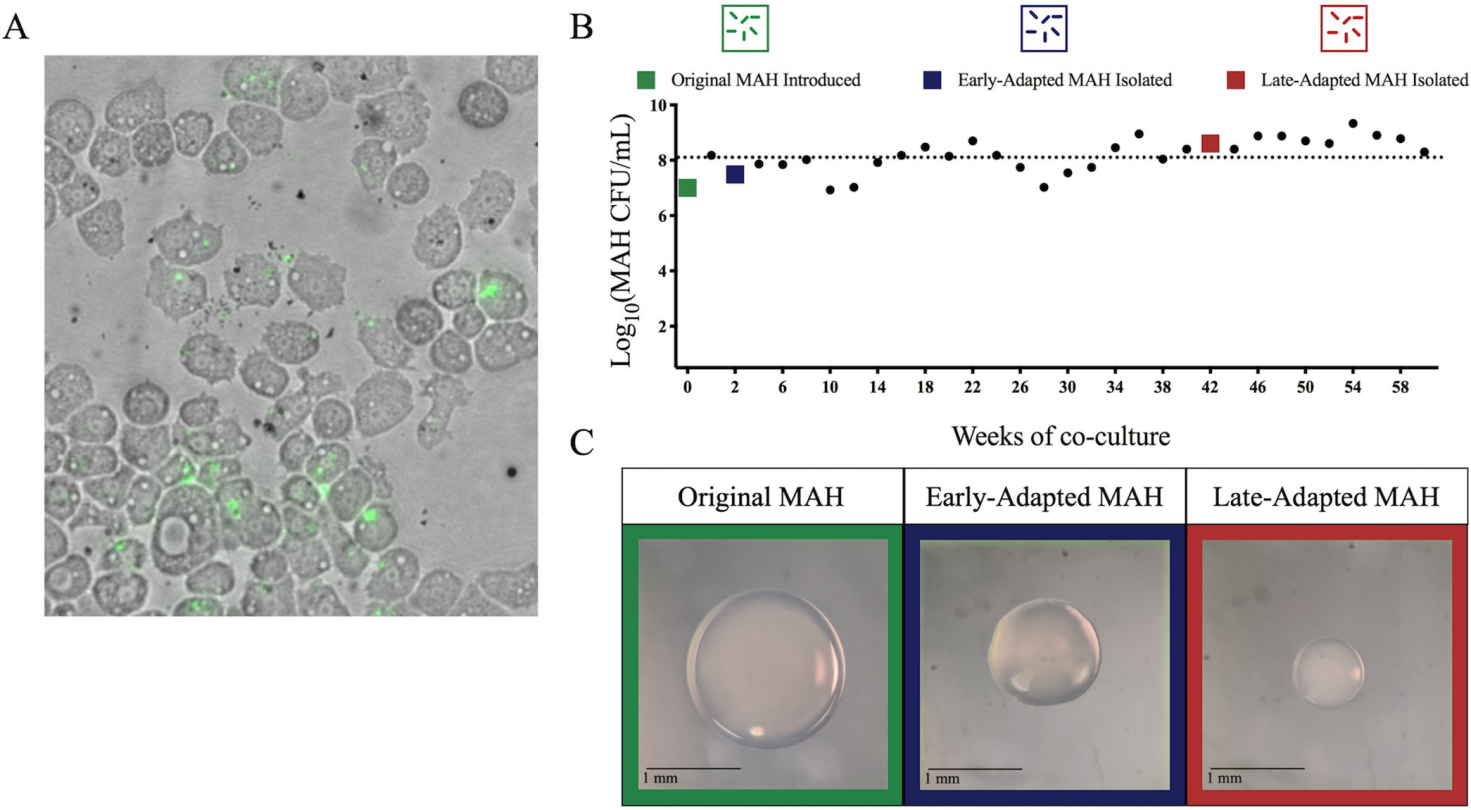
Long-term coculture of M. avium subsp. *hominissuis* and *A. lenticulata.* (A) Fluorescence microscopy image after 5 h of *A. lenticulata* and original M. avium subsp. *hominissuis* isolate coculture. (B) Original M. avium subsp. *hominissuis* isolate (green) was used to infect naive *A. lenticulata* cultures. Cell-associated CFU of M. avium subsp. *hominissuis* in *A. lenticulata* coculture were recorded every 2 weeks to monitor long-term persistence across 60 weeks. (C) At 2 and 42 weeks of coculture, the early-adapted and late-adapted isolates were recovered from coculture. Images of representative original, early-adapted, and late-adapted M. avium subsp. *hominissuis* colonies after 4 days of *A. lenticulata* coculture and ∼14 days growth on 7H10 agar plates are shown.

**FIG 2:**
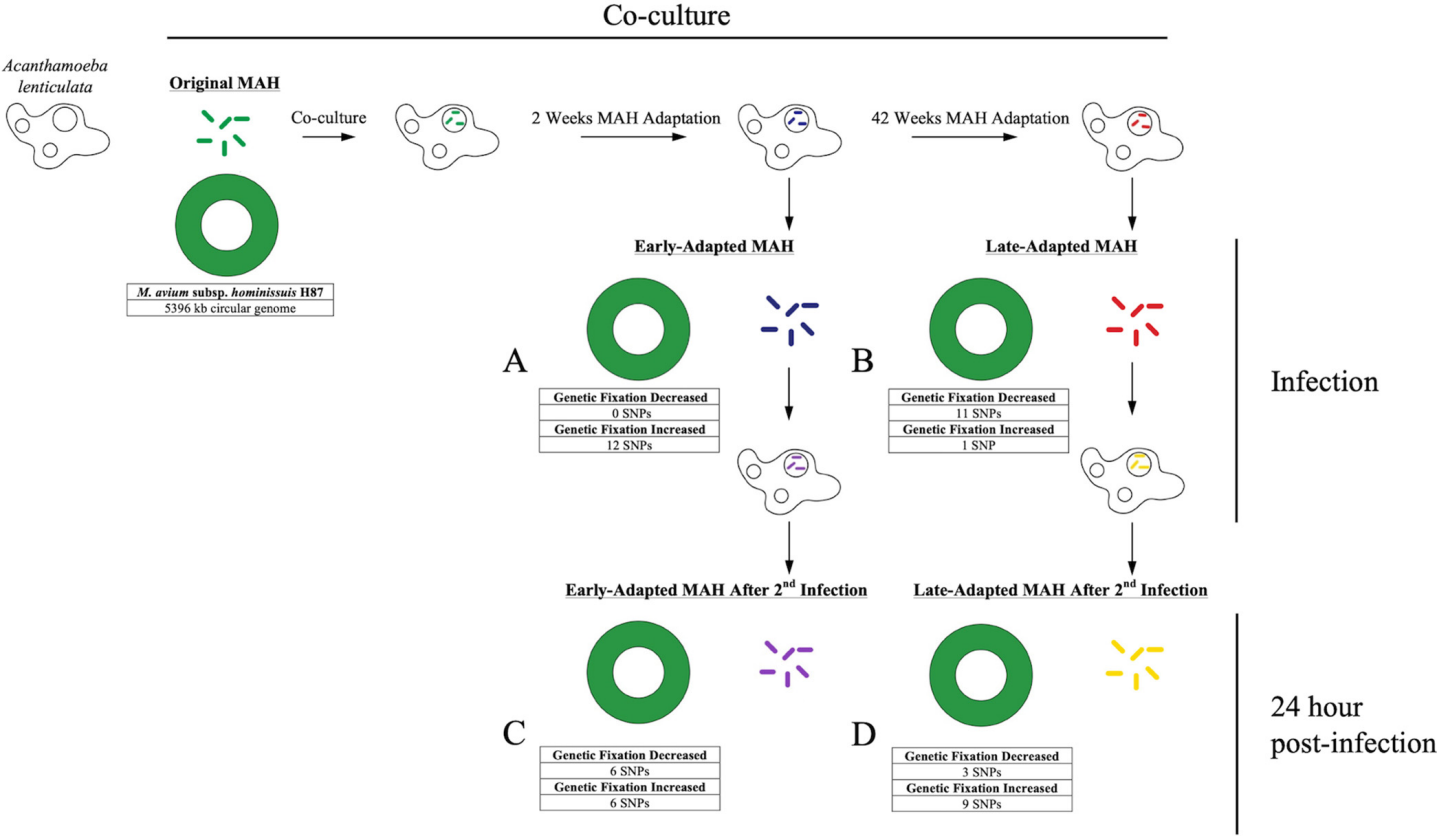
Study schematic of Mycobacterium avium subsp. *hominissuis* in *Acanthamoeba lenticulata* over time and genomic changes following coculture and reinfection in *A. lenticulata* over a 10-month period. Original M. avium subsp. *hominissuis* (green) was cocultured with *A. lenticulata* for 2 weeks or 42 weeks. Early-adapted M. avium subsp. *hominissuis* (A, blue) and late-adapted M. avium subsp. *hominissuis* isolates (B, red) were isolated after 2 and 42 weeks of coculture. Early- and late-adapted M. avium subsp. *hominissuis* were subsequently used to infect naive cultures of *A. lenticulata* and isolated after 24 h (C, purple, and D, yellow). Observed SNPs indicating increased or decreased genetic fixation (F_ST_) between stages are detailed in tables. All genomic changes incurred during coculture were determined using the original M. avium subsp. *hominissuis* strain as a reference.

Green fluorescent protein (GFP)-transformed M. avium subsp. *hominissuis* was used for long-term adaptation experiments in *A. lenticulata*. To confirm that the GFP-transformed M. avium subsp. *hominissuis* isolate was comparable to the original M. avium subsp. *hominissuis* isolate, *A. lenticulata* was cocultured with either isolate. No significant CFU differences were observed (see Fig. S1A in the supplemental material). Next, the original isolate was inoculated into coculture with *A. lenticulata* and visualized after 5 h by fluorescence microscopy (Fig. 1A). M. avium subsp. *hominissuis* burden within *A. lenticulata* cells was subsequently quantified every 2 weeks up to 60 weeks of coculture. No significant increase or decrease in the number of cell-associated M. avium subsp. *hominissuis* cells was observed over the study period (Fig. 1B). At 2 and 42 weeks following coculture, a single colony of *A. lenticulata*-adapted M. avium subsp. *hominissuis* was picked from a plate for subsequent analyses. Isolates recovered from coculture are referred to as “early-adapted” M. avium subsp. *hominissuis* (recovered 2 weeks postinfection) (Fig. 1C, blue; Fig. 2, “coculture”) and “late-adapted” M. avium subsp. *hominissuis* (recovered 42 weeks postinfection) (Fig. 1C, red; Fig. 2, “coculture”). While no significant decreases in amoeba-associated CFU were detected, a noticeable reduction in colony size with longer adaptation time in amoebae was observed by comparing the colony size of the original, early-, and late-adapted isolates (Fig. 1C; Fig. S1B). In addition, approximately 10 days were needed to achieve visible colonies of original or early-adapted M. avium subsp. *hominissuis* isolates on agar plates, whereas culture times of up to 1 month were needed to count late-adapted colonies that were significantly smaller in size, suggesting qualitatively slower growth under these culture conditions.

### WGS of original, early-adapted, and late-adapted M. avium subsp. *hominissuis* isolates.

Next, WGS was performed on 58 isolates, including six original (Fig. 2, “coculture”), 20 early-adapted, and 20 late-adapted M. avium subsp. *hominissuis* isolates (Fig. 2, “infection”), as well as six early-adapted, and six late-adapted isolates recovered 24 h postinfection in *A. lenticulata* (Fig. 2, “24 h postinfection”). Results are summarized in Table S1. One early-adapted and one late-adapted *M. avium* subsp. *hominissuis* isolate and one early-adapted M. avium subsp. *hominissuis* isolate after 24 h of infection were excluded from downstream analyses. All of the original M. avium subsp. *hominissuis* isolates were genomically identical (0 SNPs). M. avium subsp. *hominissuis* isolates with sufficient data (55/58; 94.8%) were monophyletic, showing common ancestry between the original, early-, and late-adapted M. avium subsp. *hominissuis* isolates and the original isolate (Fig. S2).

### Adaptation to *A. lenticulata* influences genomic changes in M. avium subsp. *hominissuis*.

Using WGS of 6 original, 19 early-adapted, and 19 late-adapted *M. avium* subsp. *hominissuis* isolates and 5 early- and 6 late-adapted M. avium subsp. *hominissuis* after isolates 24 h of infection, 155 SNPs were identified between the five different M. avium subsp. *hominissuis* experimental groups (i.e., original, early-, and late-adapted, and early- and late-adapted at 24 h postinfection). Among the 155 SNPs observed, 44.5% (69/155) were found in 23 genes and 55.5% (85/155) were intergenic (Table S2). The fixation index (F_ST_) was used as a measure of genetic differentiation to quantify the proportion of variance in allele frequencies among populations (i.e., original, early-adapted, and late-adapted isolates recovered from coculture and postinfection) relative to the total variance within and between isolate populations (20). Among the 69 SNPs occurring within genes, 12 SNPs (12/69 = 17.4%) in 8 genes (8/23 = 34.8%) had changes in allele frequency and showed strong genetic differentiation (F_ST_ > 0.20) between isolates recovered from *A. lenticulata* at different time points (Table 1).

**TABLE 1:** Genes containing single nucleotide polymorphisms (SNPs) with high differentiation between M. avium subsp. *hominissuis* isolates recovered from *A. lenticulata* after long-term coculture and 24 h postinfection

Locus tag	Annotation	GO category^*b*^	Coordinate^*a*^	Mean F_ST_^*a*^^,^^*b*^
BS641_RS00295	Hypothetical protein	NA	57716	0.36
			58349	0.01
			58373	0.01
			58376	0.00
BS641_RS00530	MCE family protein	Host cell penetration	107020	0.09
			107021	0.07
			107025	0.51
BS641_RS26865	Hypothetical protein	NA	967810	0.22
BS641_RS05015	Metal-dependent hydrolase	Catalysis	995112	0.24
BS641_RS08785	Acyltransferase domain-containing protein	Catalysis	1801993	0.09
			1802941	0.13
			1802944	0.05
			1802950	0.07
			1802953	0.05
			1802954	0.05
			1802955	0.07
			1802962	0.07
			1802964	0.07
			1802965	0.09
			1802969	0.09
			1802973	0.09
			1802974	0.11
			1802977	0.09
			1802983	0.09
			1802984	0.12
			1802985	0.12
			1802986	0.11
			1802994	0.11
			1802995	0.32
			1803007	0.36
			1803008	0.09
			1803009	0.36
			1803010	0.38
BS641_RS12625	Nonribosomal peptide synthetase	Catalysis	2663803	0.53
BS641_RS26605	Type II toxin-antitoxin system PemK/MazF family toxin	Transcriptional regulation	2968024	0.25
			2968025	0.20
BS641_RS20420	Nitric oxide reductase	Respiration	4355687	0.43

a Shading indicates the coordinates of each SNP that indicate high differentiation (F_ST_ ≥ 0.20) between different Mycobacterium avium subsp. *hominissuis* isolates recovered from *A. lenticulata*.

b GO, gene ontology; NA, not applicable; F_ST_, fixation index.

**FIG 3:**
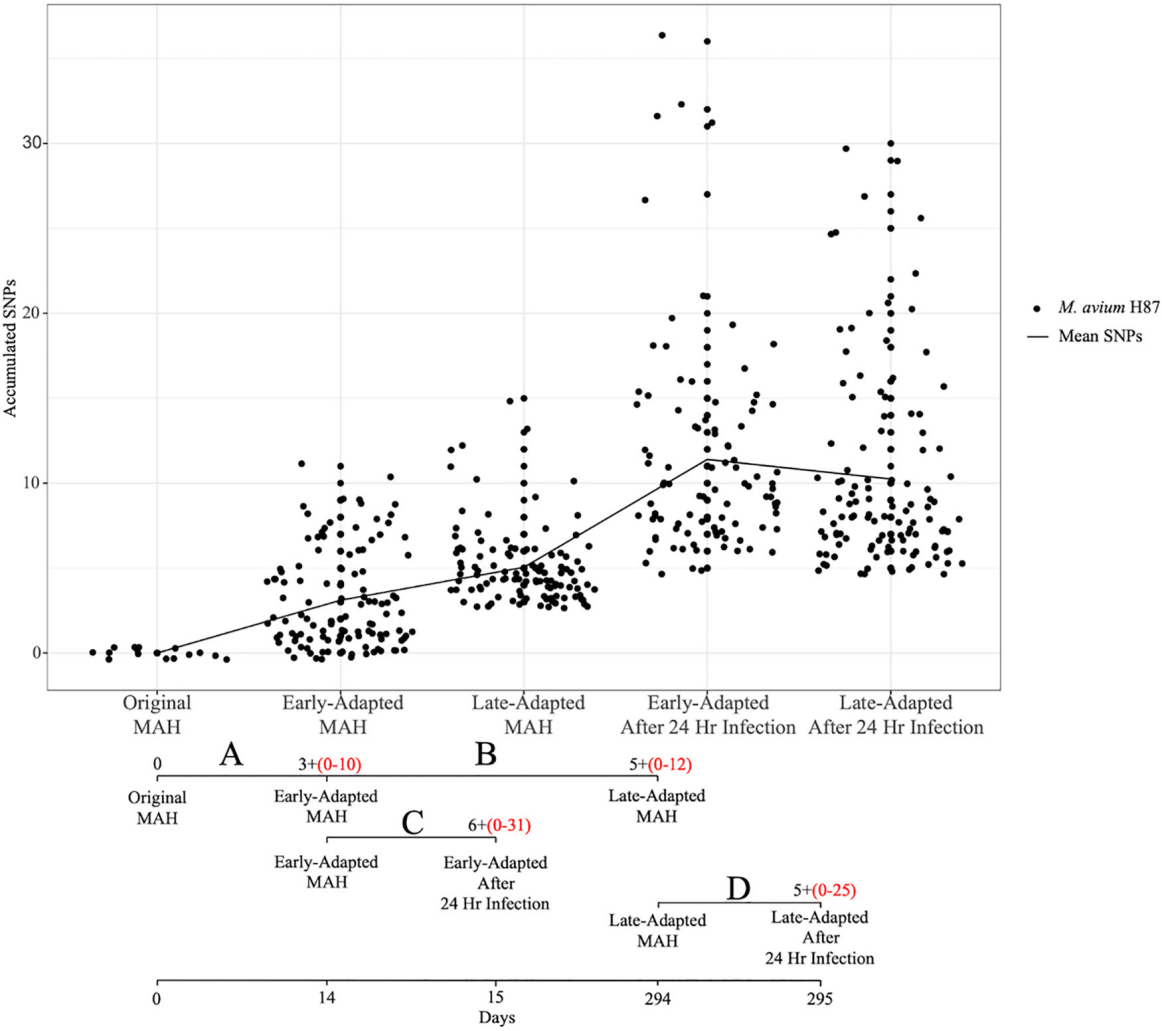
SNPs accumulated during long-term coculture. Plot of SNPs accumulated at each experimental stage using WGS information for 6 original, 19 early-adapted, and 19 late-adapted M. avium subsp. *hominissuis* isolates recovered from long-term coculture and 5 early-adapted and 6 late-adapted M. avium subsp. *hominissuis* isolates recovered at 24 h postinfection. The trend line connects the mean SNPs observed at each experimental stage. The timeline shows the experimental stages where isolates were collected for analysis and the SNPs detected at each stage (A to D). The number of SNPs represents the average mutations found in the isolates sampled at that experimental stage. SNPs in red represent the range of SNPs at the corresponding experimental stage.

Comparing M. avium subsp. *hominissuis* isolates recovered at different times across long-term coculture, we observed an accumulation of SNPs with increasing culture time. For example, when comparing the original M. avium subsp. *hominissuis* to the early-adapted isolate and the early-adapted to the late-adapted isolate, increases of 3 SNPs/isolate (0 to 10 SNPs) (Fig. 3A) and 5 SNPs/isolate (0 to 12 SNPs) (Fig. 3B) were observed, respectively. When comparing the early-adapted M. avium subsp. *hominissuis* recovered from coculture to the early-adapted isolate recovered at 24 h postinfection in *A. lenticulata*, 6 SNPs/isolate (0 to 31 SNPs) were observed (Fig. 3C). Finally, 5 SNPs/isolate (0 to 25 SNPs) were observed when comparing the late-adapted isolate to the late-adapted isolate recovered from *A. lenticulata* at 24 h postinfection (Fig. 3D). Excluding low frequency SNPs that occur in a single isolate, the progression of SNPs accumulated at a rate of 6.2 SNPs/year during coculture within *A. lenticulata*.

Because 155 SNPs were identified between the original, early-, and late-adapted M. avium subsp. *hominissuis* isolates, we investigated the possibility of microbial contamination in the *A. lenticulata* used in M. avium subsp. *hominissuis* coculture. Cell lysates of naive *Acanthamoeba* cultured contemporaneously in the long-term coculture experiment were examined by PCR amplification of 16S rRNA and mycobacterial *rpoB*. In addition to ∼900-bp amplicons representing *Acanthamoeba*, 18S rRNA, faint bands were detected at ∼478 bp, indicating the presence of bacterial 16S rRNA in naive *A. lenticulata* lysates (Fig. S3A). The same cell lysates failed to amplify using a primer set targeting mycobacterial *rpoB* (Fig. S3B) and failed to produce visible growth when used to inoculate solid and liquid culture media (Fig. S3C to E). Taken together, these results indicate the presence of nonviable, but detectable, bacteria in the *A. lenticulata* isolate used in long-term coculture with Mycobacterium avium subsp. *hominissuis*.

### *A. lenticulata* adaptation does not significantly alter M. avium subsp. *hominissuis* cell wall lipid or protein profiles.

**FIG 4:**
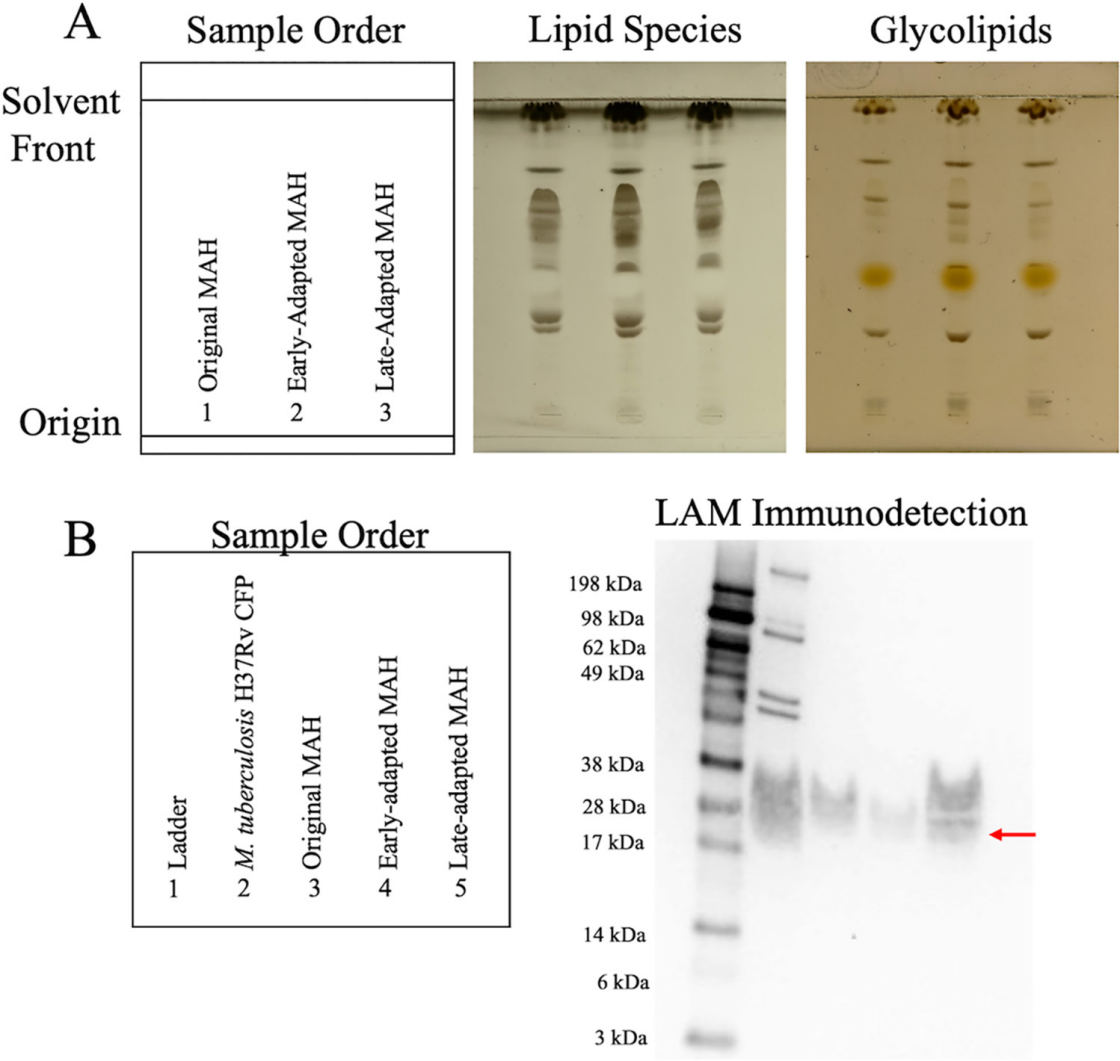
Adaptation to *A. lenticulata* does not impact M. avium subsp. *hominissuis* total lipid profiles but modulates lipoarabinomannan (LAM) expression. (A) Thin layer chromatography of total cell wall lipid species or glycolipids from original, early-, and late-adapted M. avium subsp. *hominissuis* isolates. Plates were run using 65:24:4 chloroform-methanol-H_2_O on silica plates. Total lipids were visualized with CuSO_4_ and glycolipids were visualized with α-naphthol. Samples were loaded at the origin and eluted toward the top (solvent front). Banding indicates polar (bottom) and nonpolar (top) lipids based on affinity to silica. (B) Western blotting performed on 20 μg of M. tuberculosis H37Rv culture filtrate proteins (BEI Resources NR-14825) and original, early-, and late-adapted M. avium subsp. *hominissuis* total protein lysates. Immunodetection using polyclonal M. tuberculosis anti-LAM (BEI Resources NR-13821).

To investigate the effect of long-term adaptation in amoebae on the M. avium subsp. *hominissuis* cell wall, total lipid extracts (TLE) were prepared from the original, early-adapted, and late-adapted isolates and analyzed by thin-layer chromatography (TLC). Lipid profiles showed similar banding patterns between all three isolates in terms of total lipids and glycolipids detected (Fig. 4A). In addition, TLE profiles of M. avium subsp. *hominissuis* isolates recovered at 2, 18, 30, 42, 56, and 60 weeks of coculture were evaluated, and no significant differences in total lipid and glycolipid banding patterns were observed (Fig. S4A).

As with M. avium subsp. *hominissuis* cell wall lipids, total protein profiles of the original, early-adapted, and late-adapted isolates were similar (Fig. S4B). However, a 38- to 49-kDa band was observed in the late-adapted isolate that was absent from the original and early-adapted isolates. Additionally, a second band of ∼14 kDa in the original isolate was not present in the early- and late-adapted isolates.

### Lipoarabinomannan immunodetection.

Lipoarabinomannan (LAM) is a lipoglycan found in the cell wall of mycobacteria and, among other roles in host infection, involved in the persistence in macrophages (21). To specifically probe for differences in LAM associated with amoeba adaptation, LAM immunoblotting was performed using original, early-, and late-adapted protein cell lysates. LAM was detected in all M. avium subsp. *hominissuis* isolates (Fig. 4B). However, when qualitatively compared to the original isolate, less LAM was detected in the early-adapted lysate and more LAM was detected in the late-adapted lysate.

### *A. lenticulata-*adapted M. avium subsp. *hominissuis* shows differences in intracellular persistence when used to infect naive *Acanthamoeba* and human THP-1 macrophage cultures.

**FIG 5:**
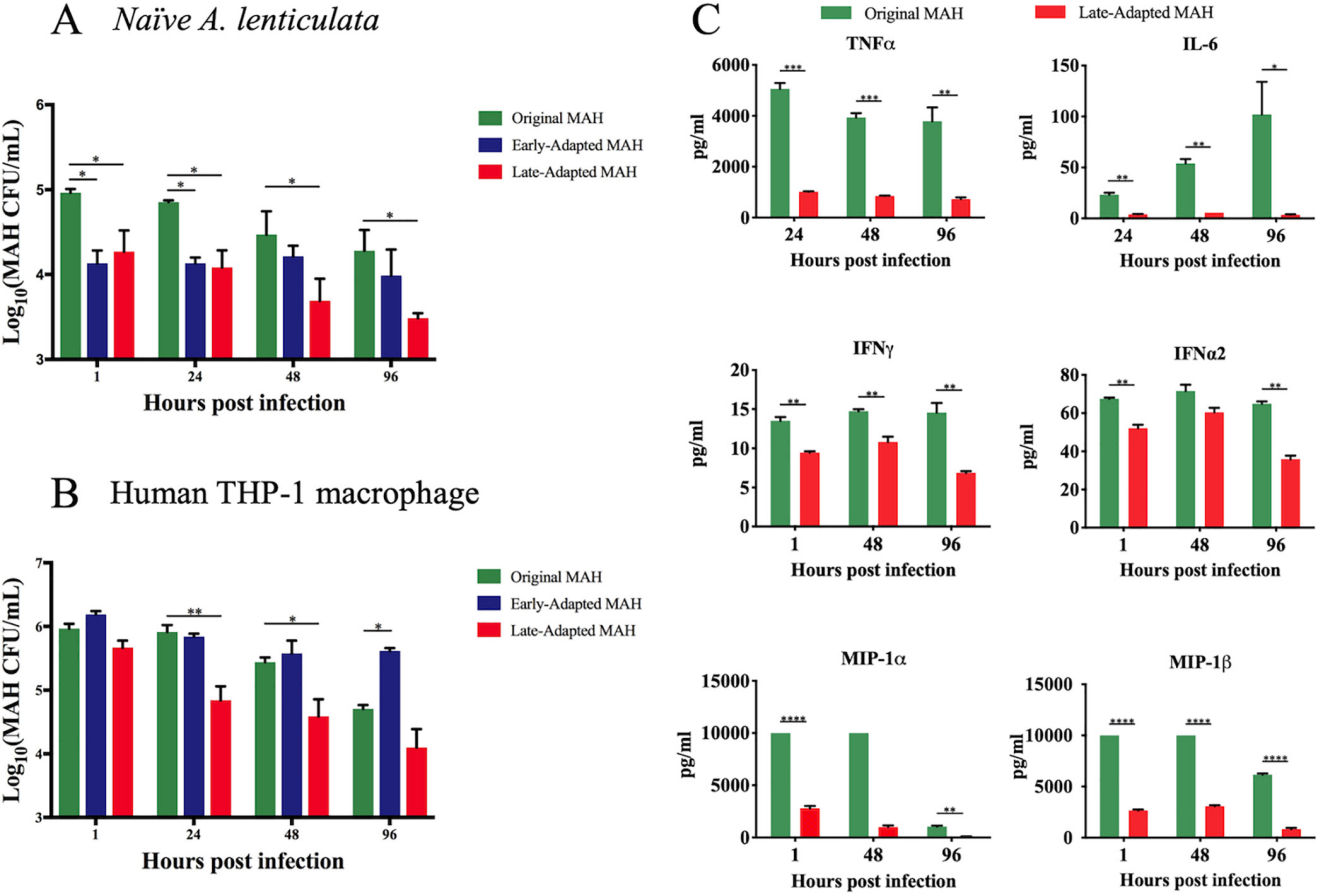
Amoeba-adapted M. avium subsp. *hominissuis* isolates show decreased survival in human macrophages and *A. lenticulata.* Original, early-adapted, and late-adapted M. avium subsp. *hominissuis* isolates were used to infect naive cultures of *A. lenticulata* (A) or human THP-1 macrophages (B) at an MOI 10:1. (C) THP-1 supernatants were used for multiplex cytokine/chemokine analyses comparing proinflammatory immune responses to the original and late-adapted M. avium subsp. *hominissuis* infection. *n* = 3 independent CFU experiments.

To determine the impact of *A. lenticulata* adaptation on the persistence of M. avium subsp. *hominissuis* within its original host, the original, early-adapted, and late-adapted isolates were used to infect naive cultures of *A. lenticulata.* At 37°C, the temperature of coculture, the numbers of both early- and late-adapted M. avium subsp. *hominissuis* were significantly reduced compared to the original isolate across 96 h (Fig. 5A). To evaluate whether the incubation temperature impacted M. avium subsp. *hominissuis* persistence in *Acanthamoeba*, as other reports have suggested (22, 23), infections were also performed in *A. lenticulata* incubated at 22°C. Similar to the 37°C data, *A. lenticulata*-adapted M. avium subsp. *hominissuis* isolates showed reduced survival when used to infect naive *A. lenticulata* compared to the original isolate (Fig. S5A). Early- and late-adapted M. avium subsp. *hominissuis* also showed reduced cell-associated viability in A. castellanii, a different *Acanthamoeba* host, compared to the original isolate (Fig. S5B).

To verify that the differences in cell-associated growth were not due to decreased viability, the original and late-adapted M. avium subsp. *hominissuis* isolates were inoculated into peptone-yeast-glucose (PYG) culture medium, independent of cells, and incubated at 22°C or 37°C. No significant changes in the growth of the two isolates were observed across 96 h in either temperature condition (Fig. S5C).

Finally, to examine the hypothesis that *A. lenticulata* adaptation enhances M. avium subsp. *hominissuis* persistence in human macrophages, the same isolates were used to infect human THP-1 cells. In this context, we considered attributes of NTM persistence to include the ability to survive exposure to THP-1 macrophages and elicit proinflammatory cytokine responses. Compared to the original isolate, the early-adapted isolate was recovered in significantly higher numbers at 96 h postinfection compared to the late-adapted isolate, which was recovered in significantly lower numbers at 24 and 48 h postinfection (Fig. 5B). As associated with reduced bacterial burden, multiplex cytokine/chemokine analyses performed on supernatants of infected THP-1 macrophages showed significantly attenuated proinflammatory cytokine (tumor necrosis factor alpha [TNF-α], interleukin 6 [IL-6], interferon gamma [IFN-γ], and IFN-α2) and chemokine (macrophage inflammatory protein 1 alpha [MIP-1α] and MIP-1β) responses to infection with the late-adapted M. avium subsp. *hominissuis* compared to the original isolate (Fig. 5C).

## DISCUSSION

One of the main goals of this study was to investigate the changes of a single M. avium subsp. *hominissuis* genotype associated with long-term persistence in *A. lenticulata* and to understand how this influences its persistence in host cells and evasion of host defenses. During the transition between early and late adaptation in amoebae, appreciable levels of SNP variation occurred in mycobacterial genes, such as those involved in catalysis, host cell penetration, respiration, and transcriptional regulation (Table 1) (24, 25). A source of genes for endosymbiotic M. avium subsp. *hominissuis* may be non-NTM DNA previously degraded by amoebae (26). Our molecular data indicate the presence of trace bacterial DNA that was not of mycobacterial origin within the uninfected *A. lenticulata* (Fig. S3). Besides mycobacteria, a number of *Proteobacteria*, *Chlamydiae*, and *Flavobacteria* are well-recognized bacterial endosymbionts of *Acanthamoeba* spp. (10, 27) and may participate in conjugal transfer between mycobacteria while living as amoebic endosymbionts (28). Yet, we cannot discount the possibility that the original infection could have been carried out with a mixture of M. avium subsp. *hominissuis* isolates despite our efforts to start with a single isolate; however, our evidence suggests at least one or more possible taxonomic sources present could have contributed to the gene pool observed over the course of long-term coculture. Further identification of the etiology of the M. avium subsp. *hominissuis* genome variation was beyond the scope of this study.

A single isolate of M. avium subsp. *hominissuis* at the early- and late-adapted time points was selected to represent the M. avium subsp. *hominissuis* population within *A. lenticulata* at these time points. While choosing one isolate to represent the population is a limitation, we were able to investigate infectivity and biochemical changes between the isolates over time. Mycobacterial cell wall lipids, specifically glycolipids, are known to influence infectivity (29, 30). TLC analyses failed to reveal differences in cell wall lipid profiles between original, early-adapted, and late-adapted M. avium subsp. *hominissuis* isolates (Fig. 4A; see Fig. S4A in the supplemental material). However, immunoblotting suggests that long-term coculture within *A. lenticulata* may increase LAM in the M. avium subsp. *hominissuis* cell wall (Fig. 4B). LAM is a highly heterogeneous and important virulence factor of mycobacteria, influencing uptake by host cells, intracellular persistence, and host immune responses (31). While further investigation into how amoeba adaptation influences M. avium subsp. *hominissuis* LAM structure and expression are warranted, our data suggest that cell wall modification may play a role in M. avium subsp. *hominissuis* persistence during amoeba adaptation.

Our observations demonstrate that M. avium subsp. *hominissuis* survives extended coculture within *A. lenticulata* of more than a year (Fig. 1B). But, by 42 weeks of adaptation in *A. lenticulata*, smaller colonies of M. avium subsp. *hominissuis* are recovered (Fig. 1C). Colony size reduction upon the transition from a free-living state to an intracellular lifestyle is well described for bacteria; however, to our knowledge, this reduction in colony size has not yet been observed in mycobacteria (26, 32–36). We also discovered that long-term adaptation in *A. lenticulata* negatively impacted two key components of mycobacterial persistence: survival within phagocytes and induction of proinflammatory cytokine responses (37–41). The bacterial burden of both original and late-adapted M. avium subsp. *hominissuis* decreased over the course of infection in THP-1 macrophages (Fig. 5B). Similar to previous findings (42), cytokine analyses performed on the supernatants collected from the infected THP-1 cells showed significantly less production of proinflammatory cytokines by THP-1 cells in response to the late-adapted M. avium subsp. *hominissuis* isolate compared to the original M. avium subsp. *hominissuis* isolate (Fig. 5C), illustrating attenuation of the proinflammatory response to M. avium subsp. *hominissuis* after long-term adaptation to *A. lenticulata*. High levels of TNF-α and IFN-γ may explain this reduction in CFU in original M. avium subsp. *hominissuis* infection; however, the absence of these host-protective cytokines in late-adapted M. avium subsp. *hominissuis* infection fails to explain trends in CFU (43, 44). These data could demonstrate that adaptation in amoebae over long-term coculture reduces the ability of M. avium subsp. *hominissuis* to replicate and induce host-protective proinflammatory cytokines. However, a more comprehensive analysis of the phenotypic changes should be performed, including characterizing the anti-inflammatory responses by measuring IL-4 and IL-10 levels, as well as other host defense mechanisms, including apoptosis, autophagy, and phagosome-lysosome fusion. Because we were unable to measure cytokines produced after infection with the early-adapted M. avium subsp. *hominissuis* isolate at the time, we do not know if these responses would mirror the trends observed for the original or late-adapted M. avium subsp. *hominissuis* isolates.

This work was originally initiated to test the hypothesis that M. avium subsp. *hominissuis* infectivity in human macrophages increases with longer “training” time in *A. lenticulata* (14, 45). However, the opposite was observed in our data and deviated from previous work, which showed increased M. avium subsp. *hominissuis* replication within human monocyte-derived macrophages (MDM) after a 10-day adaptation within *Acanthamoeba* (15). However, our study differs in the following parameters: (i) use of a cell line compared to primary cell cultures, (ii) longer length of time for adaptation in amoebae, and (iii) culturing of the amoeba-adapted isolates and using stocks for infections, whereas previous work used M. avium subsp. *hominissuis* immediately recovered from amoebae in MDM infections. Importantly, while *in vitro* macrophage infection is a well-established model for assaying mycobacterial infections, future investigation of *in vivo* host responses to amoeba-adapted Mycobacterium avium subsp. *hominissuis* will be needed to better characterize the impacts of long-term coculture on the ability of NTM to infect macrophages. Yet, our work suggests the long-term persistence and endosymbiosis between M. avium subsp. *hominissuis* and *A. lenticulata* achieved in our study can occur in the environment, and thus elevates the suspicion that environmental amoebae act as natural reservoirs for M. avium subsp. *hominissuis*. We posit that it is the ability of amoebae to act as a vector for M. avium subsp. *hominissuis* transmission and persistence, not reduced M. avium subsp. *hominissuis* virulence, which may contribute, in part, to the increasing prevalence of NTM pulmonary infection. Most patients infected with M. avium subsp. *hominissuis* are immunocompromised and susceptible to infection from opportunistic pathogens, so the reduced phenotypes we observed over time is likely not affecting the rate of NTM disease.

Despite the use of different hosts (i.e., amoebae compared to humans), a portion of our study results align with Kannan et al., who showed rapid SNP evolution in M. avium isolates from chronically infected patients and studied the changes in infectivity over time (42). This study revealed higher mutation rates of M. avium during persistent infections and infection of murine macrophages with sequentially collected patient isolates showing downregulation of inflammatory cytokines by host-adapted strains. This group proposed that genetic variation throughout the course of infection might be due to microevolution of the founding strain following the infection, that each patient’s infecting inoculum might have contained mixed strains, or that each patient could have been reinfected with different strains at different times. Instead of an unknown inoculum, our experimental design used a well-characterized isolate of M. avium subsp. *hominissuis* that was transformed with *gfp* to infect a variety of host cells. In our work, we found the core genome variation clearly showed that the original, early-, and late-adapted M. avium subsp. *hominissuis* isolates collected from the lysed *A. lenticulata* share most recent common ancestry, diminishing the likelihood of mixed isolates or reinfection with diverse strains. The observed mutation rate of our M. avium subsp. *hominissuis* isolate in amoebae was 6.2 SNPs/year and this is within the range of the observed mutation rates from the patient M. avium subsp. *hominissuis* isolates from the Kannan study, at 4.5 to 7.1 SNPs/year. Because we also observed reduced levels of proinflammatory cytokines by THP-1 macrophages in response to late-adapted M. avium subsp. *hominissuis*, we concur with Kannan et al. that host adaptation may influence the inflammatory properties of M. avium.

Together, our data illustrate that long-term adaption in *A. lenticulata* negatively impacts M. avium subsp. *hominissuis* genomic change, colony morphology, and persistence. Prior to the present study, WGS and population genomics had rarely been used to study the evolution of M. avium subsp. *hominissuis* over time in amoebae. We believe it will be just as important in future work to understand how M. avium subsp. *hominissuis* impacts the *A. lenticulata* genome and define the amoeba-specific innate immune responses produced in response to NTM infection. However, WGS of the much larger and more complex genome of *A. lenticulata* was beyond the scope of the current study. Further studies into the underpinning mechanism(s) responsible for reduced survival of M. avium subsp. *hominissuis* in human macrophages with longer adaptation time in amoebae are needed to increase our understanding of the complex molecular relationships and genetic exchanges that exist and take place between NTM and environmental protozoa.

## MATERIALS AND METHODS

### M. avium subsp. *hominissuis*, *Acanthamoeba*, and human macrophage cultures.

We isolated M. avium subsp. *hominissuis* strain H87 from an indoor sink faucet (6) and reported its complete genome, NCBI accession number NZ_CP018363 (46). This parent isolate was stably transformed via electroporation with a green-fluorescent protein (GFP) expression plasmid to create a fluorescently labeled isolate used in subsequent *Acanthamoeba* coculture and referred herein as the “original” M. avium subsp. *hominissuis* isolate.

*Acanthamoeba lenticulata* ATCC 30841 and Acanthamoeba castellanii trophozoites were cultured in sterile peptone-yeast-glucose (PYG) broth at 22°C and 37°C for maintenance cultures. To prevent amoeba overgrowth and encystment, maintenance cultures were centrifuged to pellet the amoebae, spent medium was removed, and fresh PYG medium was added every fourth passage day. Human THP-1 monocytes were cultured and differentiated into macrophages as previously described (47, 48).

### Visualization of M. avium subsp. *hominissuis* in amoebae by fluorescence microscopy.

*A. lenticulata* cells (5 × 10^4^) were suspended in 500 μl of PYG medium and seeded into wells of a Nunc Lab-Tek II chamber slide (Thermo Fisher Scientfic, Waltham, MA). Slides were incubated at 37°C overnight and infected with a multiplicity of infection (MOI) of 10 original M. avium subsp. *hominissuis* cells per amoeba. After 5 h of infection, spent medium was removed and the monolayer was washed twice with phosphate-buffered saline (PBS) and fixed using 4% paraformaldehyde. Fixing medium was aspirated and the monolayer was washed with PBS. Slides were immediately visualized using fluorescence microscopy.

### Long-term coculture of *A. lenticulata* with M. avium subsp. *hominissuis*.

*A. lenticulata* cultures were adjusted to a concentration of 1 × 10^6^ amoebae/ml in fresh PYG and original M. avium subsp. *hominissuis* stock culture was added to achieve an MOI of 1:1. The coculture was incubated at 37°C and spent medium was removed and replaced every 2 weeks with fresh PYG for up to 60 weeks following coculture. At every medium change, the persistence of M. avium subsp. *hominissuis* within *A. lenticulata* was monitored by transferring 10 ml of coculture into a conical vial and centrifuging at 771 × *g* for 5 min at room temperature. To recover amoeba-associated M. avium subsp. *hominissuis*, cell pellets were lysed by adding 500 μl 0.01% Triton X-100 (Sigma, St. Louis, MO) and an equal volume of Middlebrook 7H9 plating solution (7H9, oleic acid-albumin-dextrose-catalase [OADC] enrichment, Tween 80). Lysates were serially diluted, plated in duplicate on Middlebrook 7H10 agar, and incubated for 2 weeks at 37°C, and the CFU were counted.

On two occasions (i.e., at 2 and 42 weeks of coculture), a colony of *A. lenticulata*-adapted M. avium subsp. *hominissuis* was picked from CFU plates and subcultured in 7H9 broth and incubated under rotation at 150 rpm at 37°C for 2 weeks to obtain stock cultures. These isolates are referred to throughout this study as “early-adapted” M. avium subsp. *hominissuis* (2 weeks of coculture) and “late-adapted” M. avium subsp. *hominissuis* (42 weeks of coculture). Serial dilutions were plated on 7H10 agar to determine the starting stock CFU. M. avium subsp. *hominissuis* stocks were stored at –80°C until use. Early- and late-adapted M. avium subsp. *hominissuis* were used to infect naive cultures of *A. lenticulata* (MOI 10:1) for 24 h. Amoebae were lysed and serial dilutions of the lysate were plated onto 7H10 agar to quantify CFU. After 2 weeks of incubation, six early-adapted (see Table S1, 1 through 6, in the supplemental material) and late-adapted (Table S1, 1 through 6) M. avium subsp. *hominissuis* colonies were picked from CFU plates and grown in 7H9 broth for sequencing.

To semiquantify changes in the size of colonies recovered after amoeba infection, diameters of representative original, early-adapted, and late-adapted M. avium subsp. *hominissuis* colonies plated on 7H10 agar were measured using a Laxco SeBa Pro4B microscope at 4× magnification. Twenty colonies per isolate were measured and averaged.

### M. avium subsp. *hominissuis* gDNA extraction.

Prior to genomic DNA (gDNA) extraction, 20 isolated colonies of early-adapted, 20 colonies of late-adapted, and 6 colonies of the original isolate (Table S1) were picked and grown in 7H9 broth and high-quality genomic DNA (gDNA) was isolated (49). gDNA was also isolated from early- and late-adapted M. avium subsp. *hominissuis* at 24 h after infection in *A. lenticulata* to determine the effect of reintroduction to amoebae on the M. avium subsp. *hominissuis* genome.

### M. avium subsp. *hominissuis* WGS, genomic analyses, and gene annotation.

To generate WGS, sequencing libraries were prepared using the Nextera DNA FLEX library preparation kit (Illumina) and sequenced on an Illumina MiSeq using 2 × 300 paired-end chemistry. Isolate metadata, NCBI Short Read Archive accession, and Bioproject PRJNA587788 accession numbers are detailed in Table S1. Illumina sequence data were analyzed through an in-house workflow. Read files were trimmed of adapter sequences and bases under quality thresholds (q-score < 20) using Skewer (50). Quality-trimmed read files were mapped to the M. avium H87 reference genome to identify SNPs using Bowtie2 (51) and postprocessed using SAMtools (52), outputting SNPs to variant call format (VCF) files. Each isolate’s VCF file was filtered, removing SNPs without at least four FASTQ reads coverage (DP4 ≤ 4) and at least 75% of basecalls supporting the reference or alternative allele (AF ≤ 0.75). A phylogenetic tree was created using RAxML (53), the GTR model and 500 bootstrap replicates.

Population differentiation was identified using the fixation index (F_ST_), a measure of population differentiation wherein 0 indicates no differentiation and a value of 1 implies populations have fixed alternative allelic states and are completely differentiated. F_ST_ was calculated at observed SNPs using vcftools v0.1.17 (54) with haploid mode enabled (https://github.com/vcftools/vcftools/pull/69).

Quality-trimmed reads were used to *de novo* assemble the genomes of each isolate using Unicycler (55) and resultant assembly contigs were reordered against the H87 reference genome using MAUVE (56). The reordered *de novo* assemblies were annotated using Prokka (57) and analyzed by Roary (58). BLAST analyses were used to compare the translated nucleotide sequences against nonredundant NCBI protein sequences and to annotate hypothetical proteins.

To calculate the SNP accumulation rate during coculture within *A. lenticulata*, the mean difference between the early-adapted and late-adapted M. avium subsp. *hominissuis* was calculated to be 4.99 SNPs/294 days (days between the early- and late-adapted M. avium subsp. *hominissuis*). To extrapolate the annual mutation rate, the following equation was used: 4.99 SNPs/294 days = *x* SNPs/365 days, equaling 6.2 SNPs per year.

### 16S and *rpoB* amplification.

DNA was extracted from *A. lenticulata* (49) with the inclusion of a bead beating step. DNA concentrations were normalized based on Qubit fluorescent quantitation, and 0.8 ng of each template was used for PCR in duplicate. For 16S amplification, adapter-modified Earth Microbiome Project 16S PCR primers 515F and 926R were used (59). A second mycobacterium-specific amplification used primers against *rpoB* (60) and resulted in an expected band of 831 bp if mycobacteria were present. Amplifications were performed using LA *Taq* (TaKaRa) optimized for high GC content, with the following reaction conditions: for 16S, 94°C for 3 min, 28 cycles of amplification (15 s at 94°C, 30 s at 55°C, and 30 s at 72°C), and extension of 5 min at 72°C; for *rpo*, 94°C for 3 min, 35 cycles of amplification (15 s at 94°C, 30 s at 64°C, and 90 s at 72°C), and extension of 5 min at 72°C. PCR products were separated by electrophoresis on a 1.8% agarose gel with GelRed (Phoenix Research Products) in 1× Tris-acetate-EDTA at 80 V for 75 min and visualized using a Bio-Rad gel imager on the UV setting.

### Microbial assessment of uninfected *A. lenticulata*.

Periodic monitoring of uninfected *A. lenticulata* at weeks 2, 13, and 42 was conducted by lysing *A. lenticulata* cells in 200 μl of 0.5% SDS. The lysate was neutralized by adding 200 μl of plating solution and 50 μl was spread plated onto two Trypticase soy agar (TSA) plates incubated at either 37°C or 22°C. The remaining lysate was inoculated into Trypticase soy broth (TSB) and the volume was divided for continued incubation at either 37°C or 22°C for up to 10 days.

### M. avium subsp. *hominissuis* lipid analyses.

Aliquots of Proskauer-Beck culture medium (50 ml) were inoculated with M. avium subsp. *hominissuis* isolates and incubated at 37°C for up to 1 month. Once turbid, the cultures were centrifuged at 557 × *g* for 60 min, supernatants were removed, and the pellets were stored at –80°C. For total lipid extraction, thawed pellets were resuspended in 1:1 chloroform-methanol and rocked for 2 h. Samples were centrifuged at 2,637 × *g* for 10 min; then 4 ml of the supernatant was removed, and 4 ml of 1:1 chloroform-methanol was added back to each sample. These steps were repeated two additional times and the supernatants from each fraction were pooled. After each pooling, the solvent was evaporated under N_2_. Samples were resuspended in 2:1 chloroform-methanol and 15 μl was spotted onto duplicate silica plates for analysis by TLC using a 65:24:4 chloroform-methanol-H_2_O solvent system. Plates were sprayed with CuSO_4_ to visualize total lipids or α-naphthol to visualize glycolipids and developed under dry heat.

### Lipoarabinomannan Western blotting.

M. avium subsp. *hominissuis* isolates were cultured for 2 weeks in 7H9 broth. Once turbid, the cultures were centrifuged at 5,000 × *g* for 10 min, supernatant was removed, and pellets were stored at –80°C. Extraction of M. avium subsp. *hominissuis* total proteins was performed using B-PER (Thermo Fisher Scientific, Waltham, MA) supplemented with DNase I and lysozyme (Thermo Fisher Scientific). Twenty micrograms of M. avium subsp. *hominissuis* total proteins and Mycobacterium tuberculosis strain H37Rv culture filtrate proteins (BEI Resources NR-14825) was separated by SDS-PAGE on a 4 to 12% Bis-Tris–morpholineethanesulfonic acid (MES) gel (Thermo Fisher Scientific) and transferred onto a polyvinylidene difluoride (PVDF) membrane (iBlot, Invitrogen). Membranes were blocked in 5% nonfat milk-PBS with 0.25% Tween 20 (PBST) overnight at 4°C. Polyclonal anti-LAM primary antibody (BEI Resources NR-13821) was diluted 1:1,000 in 5% nonfat milk in PBST and used with 1:1,000 horseradish peroxidase (HRP)-conjugated goat anti-rabbit secondary antibody (Thermo Fisher Scientific). NR-14825 and 13821 reagents were obtained through BEI Resources, NIAID, and the NIH. Blots were visualized using SuperSignal West Femto maximum sensitivity substrate (Thermo Fisher Scientific) on the iBright CL1000 imager (Invitrogen, Carlsbad, CA).

### Infection of naive *Acanthamoeba* or THP-1 macrophages cultures with amoeba-adapted M. avium subsp. *hominissuis*.

To study the possibility that amoeba-adapted M. avium subsp. *hominissuis* is better suited to infect the same host when encountered again, “early-adapted “and “late-adapted” M. avium subsp. *hominissuis* isolates were used to infect naive cultures of *A. lenticulata*. After 1, 24, 48, and 96 h of infection, the cultures were lysed and plated onto 7H10 agar to recover colonies. THP-1 monocytes were seeded at 1 × 10^6^ cells/ml into 6-well plates with 0.1 μg/ml phorbol 12-myristate 13-acetate (PMA) (Sigma-Aldrich, St. Louis, MO) and incubated at 37°C in 5% CO_2_ for 48 h to facilitate differentiation into macrophages. The incubation temperature of 37°C was chosen to most closely mimic M. avium subsp. *hominissuis* endosymbiosis during human infection; prior studies found no significant different between 37°C and 22°C (61–64). At the time of infection, the spent medium was removed and the cells were washed with PBS and infected with 1 × 10^7^ M. avium subsp. *hominissuis*/ml (MOI, 10:1) for 1 h. Unphagocytosed M. avium subsp. *hominissuis* cells were removed by aspiration, washed, and replaced with fresh medium. Cell-associated M. avium subsp. *hominissuis* cells were recovered at 1, 24, 48, and 96 h postinfection by the addition of equal volumes of 0.025% sodium dodecyl sulfate (SDS) (Sigma-Aldrich, St. Louis, MO) and 7H9 plating solution. Serial dilutions of the lysate were plated in duplicate and incubated at 37°C for up to 21 days. Conditioned medium collected throughout the THP-1 experiments was used for cytokine and chemokine analysis using the Multiplex Map kit (Millipore, MA) performed by the National Jewish Health Complement Laboratory.

### Statistical analyses.

The data were analyzed with GraphPad 8.0 using two-way ANOVA to determine statistical significance. For CFU data, all analyses compared the early-adapted and late-adapted M. avium subsp. *hominissuis* to the original isolate as a control. Values with *P* of <0.05 were considered statistically significant. Data are expressed as means ± standard error of the mean (SEM) for three or more independent experiments.

## Supplementary Material

Supplemental file 1
