# Supplementary material for: Measurable Genomic Changes in Mycobacterium avium subsp. hominissuis after Long-Term Adaptation in Acanthamoeba lenticulata and Reduced Persistence in Macrophages
Source: J Bacteriol. 2021 Feb 22;203(6):e00257-20. doi: 10.1128/JB.00257-20 (PMC8095452; doi:10.1128/JB.00257-20)
Supplement: Supplemental file 1 [file JB.00257-20-s0001.pdf]

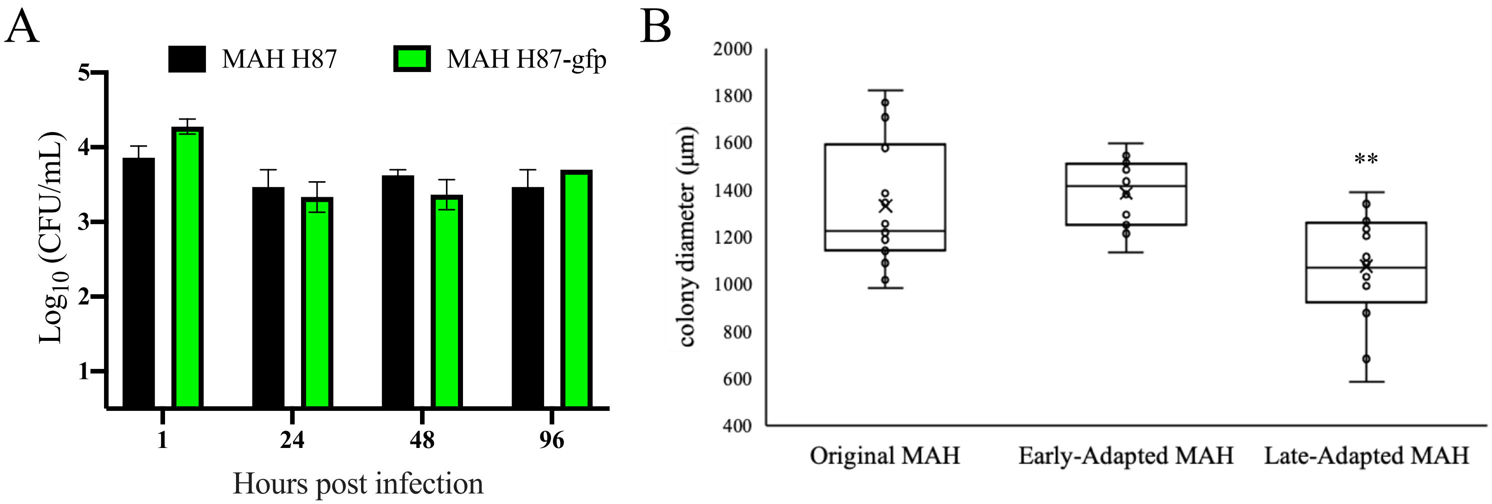

Figure S1: No significant difference in the growth of original MAH H87 and MAH H87-gfp in *A. lenticulata,* but significant reduction in MAH colony size after long-term co-culture in *A. lenticulata.* **(A)** Original MAH H87 (black) and green fluorescent protein (gfp) labeled MAH H87 (green) were used to infect *A. lenticulata* incubated at 22 °C (MOI 10:1). CFU were quantified at 1, 24, 48 and 96 hours post infection. n= 3 independent experiments. **(B)** Average colony diameter of original (left), early-adapted (middle) and late-adapted MAH colonies were measured on a Laxco SeBa Pro4B microscope at 4X magnification. Significance determined as compared to original MAH. n=20 individual colonies measured per isolate.

Figure S1

Figure S2

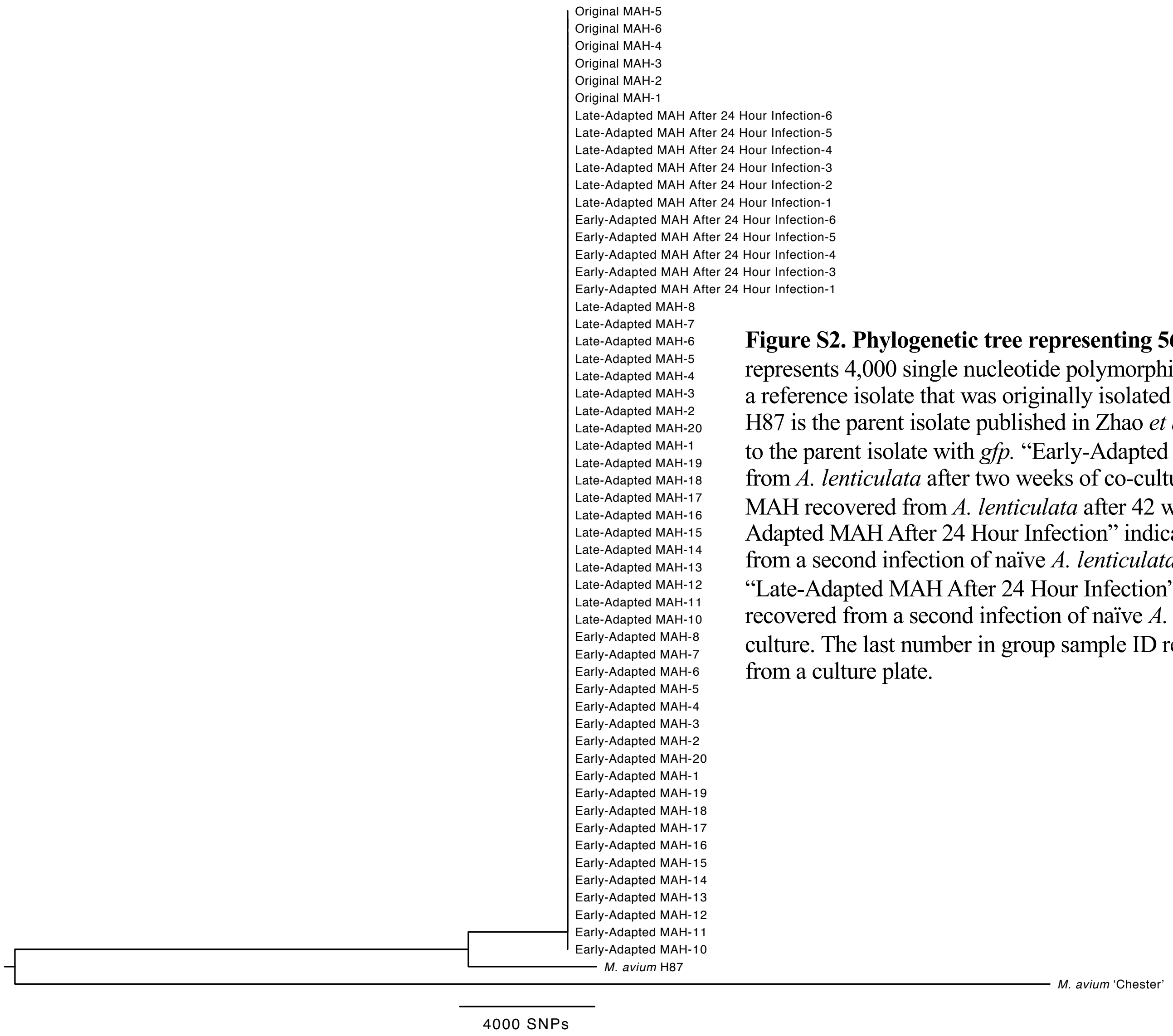

Figure S2. Phylogenetic tree representing 56 MAH H87 isolates. Scale bar represents 4,000 single nucleotide polymorphisms (SNPs). *M. avium* 'Chester' is a reference isolate that was originally isolated from an infected hen. *M. avium* H87 is the parent isolate published in Zhao *et al*., 2017. "Original MAH" refers to the parent isolate with *gfp.* "Early-Adapted MAH" refers to MAH recovered from *A. lenticulata* after two weeks of co-culture. "Late-Adapted MAH" refers to MAH recovered from *A. lenticulata* after 42 weeks of co-culture. "Early-Adapted MAH After 24 Hour Infection" indicates early-adapted MAH recovered from a second infection of naïve *A. lenticulata* after 24 hours of co-culture. "Late-Adapted MAH After 24 Hour Infection" refers to late-adapted MAH recovered from a second infection of naïve *A. lenticulata* after 24 hours of co-culture. The last number in group sample ID refers to the colony number picked from a culture plate.

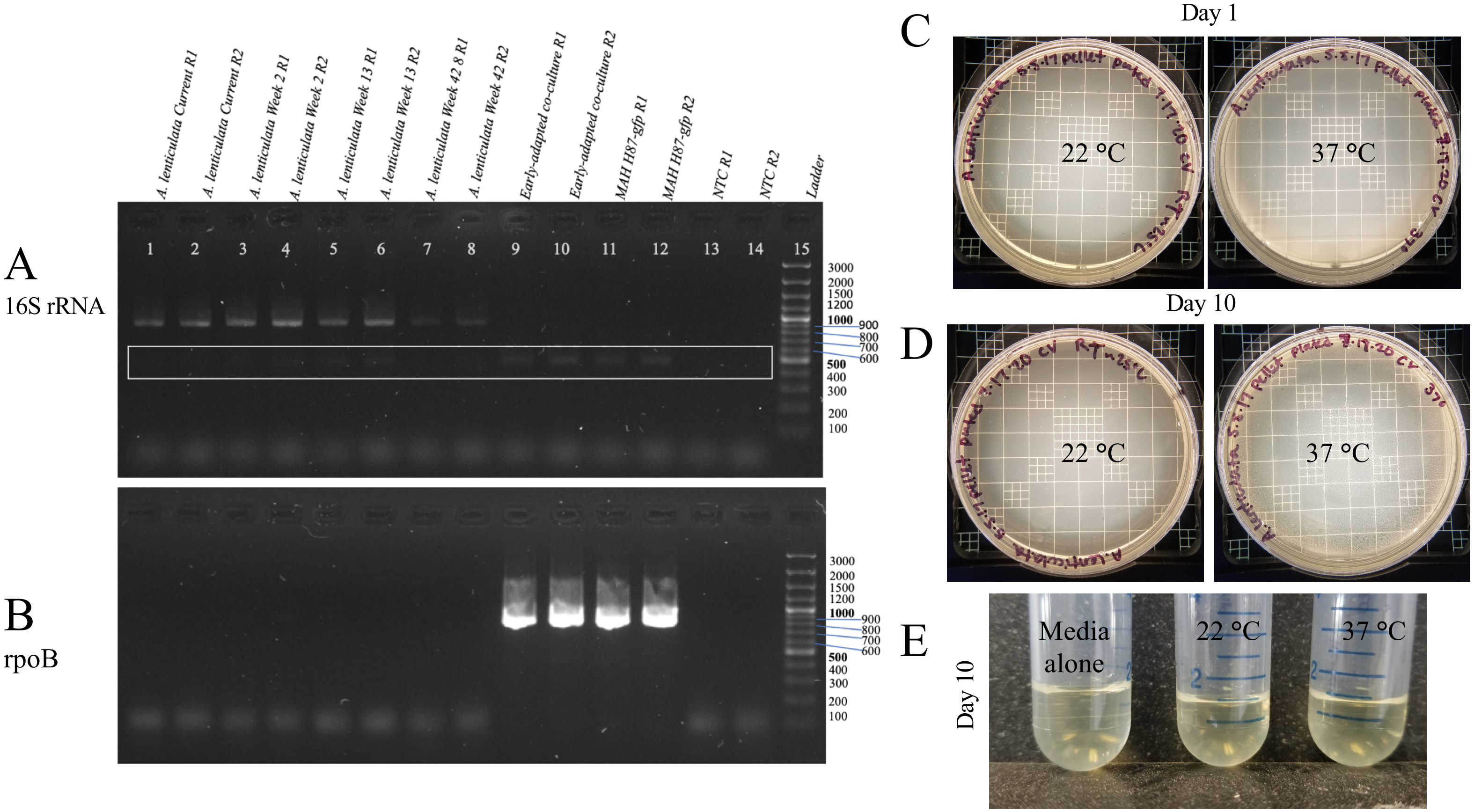

Figure S3

Figure S3. 16S rDNA and mycobacteria-specific rpoB PCR of *A. lenticulata* and culture testing. (A) 16S rRNA PCR gel electrophoresis of uninfected *A. lenticulata* collected at weeks 2, 13, 42 along with a sample from a current *Acanthamoeba* culture. DNA was extracted from an early-adapted (2 week) *A. lenticulata* culture infected with *M. avium* subsp. *hominissuis* and *M. avium* subsp. *hominissuis* only DNA was also included as controls. NTC = no template control. Each sample was run in duplicate ("R1 or R2"). Eukaryotic DNA ~ 900 bp. Bacterial 16S ~ 478 bp; area highlighted by white box. **(B)** Mycobacteria-specific rpoB PCR gel electrophoresis the same samples detailed in "A." NTM rpoB ~ 831 bp. **(C)** *A. lenticulata* parent culture (before MAH infection) lysate were spread plated on TSA plates and incubated at 22 °C or 37°C. Plates were observed each day for viable growth up to 10 days **(D)**. **(E)** The remaining lysate was inoculated into TSB and incubated at 22 °C or 37 °C. Turbidity was monitored up to 10 days. Images shown were taken immediately after vortexing the cultures for 10 seconds.

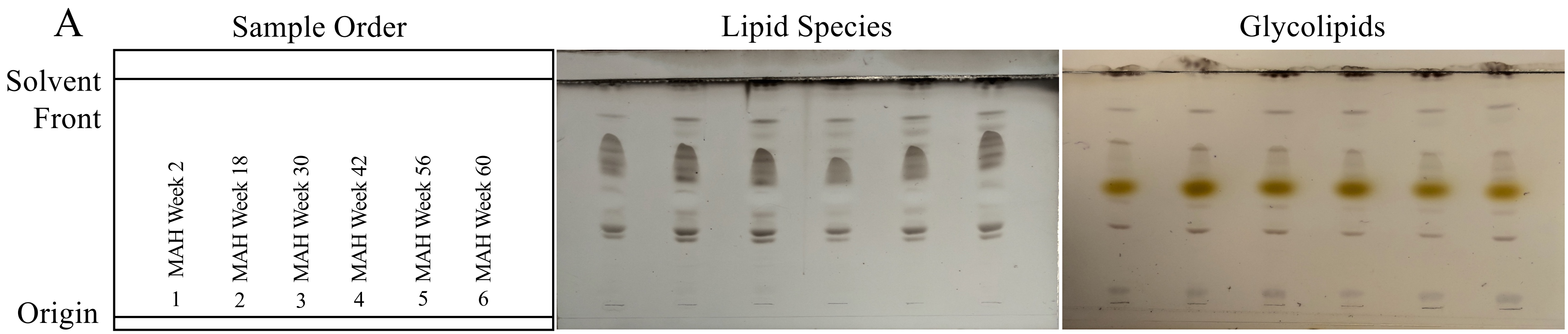

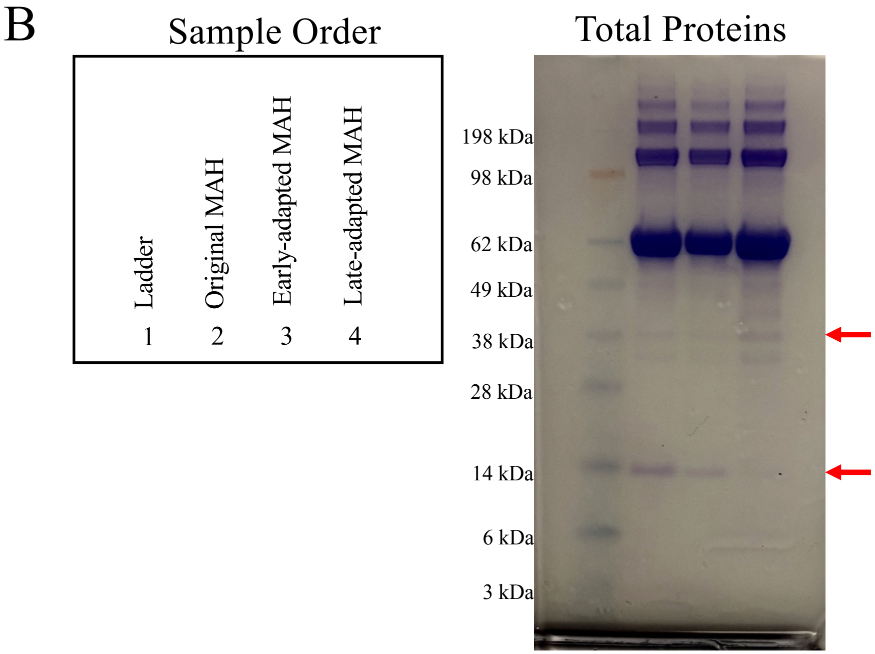

Figure S4: Adaptation to *A. lenticulata* does not affect MAH cell wall lipid or protein profiles. (A) TLC of total cell wall lipid species or glycolipids from original, early- and late-adapted MAH isolated across one year of *A. lenticulata* co-culture. Plates were run using 65:24:4 chloroform:methanol:H_2O on silica plates. Total lipids were visualized with $CuSO_4$ (lipid species) or α-naphthol (glycolipids). Samples were loaded at origin eluted towards the top (solvent front). Banding indicates polar (bottom) and nonpolar (top) lipids based on affinity to silica. **(B)** SDS-PAGE and Imperial™ protein stain (Invitrogen) performed on 20 µg of total protein isolated from original, early-adapted and late-adapted MAH. Red arrows indicate regions in which protein profiles differ between MAH isolates.

Figure S4

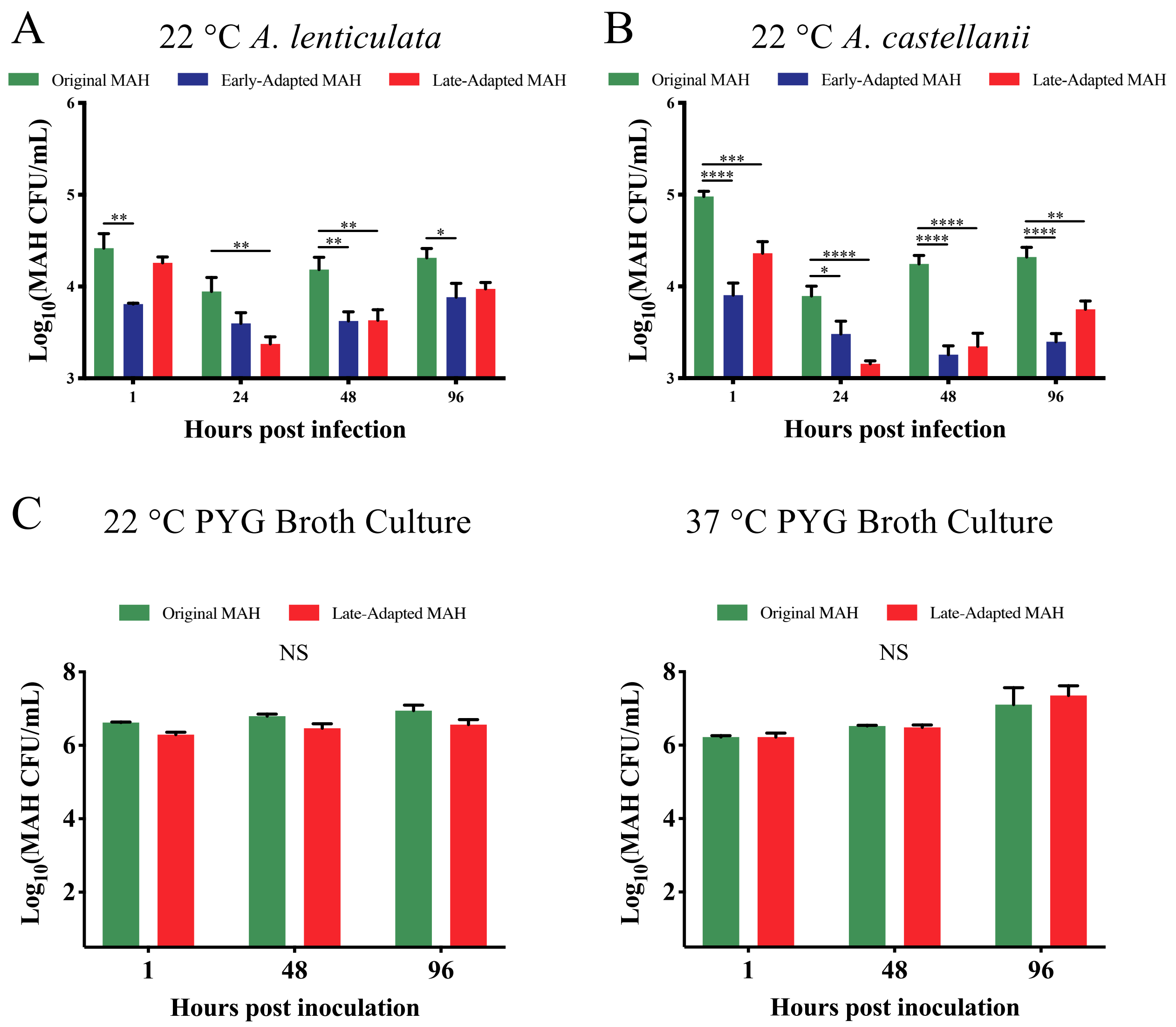

Figure S5: Reduced survival of MAH in a different *Acanthamoeba* species, but no difference at 22 ˚C incubation. Original, early-adapted and late-adapted MAH were used to infect naïve cultures of **(A)** *A. lenticulata* or **(B)** *A. castellanii* incubated at 22 °C. **(C)** 1x10^{7} CFU of original and late-adapted MAH were inoculated into PYG broth incubated at 22 °C (left) or 37 °C (right) for 96 hours.

Figure S5

Supplemental Figure Legends

Figure S1: No significant difference in the growth of original MAH H87 and MAH H87-gfp in *A. lenticulata,* but significant reduction in MAH colony size after long-term co-culture in *A. lenticulata.* (A) Original MAH H87 (black) and green fluorescent protein (gfp) labeled MAH H87 (green) were used to infect *A. lenticulata* incubated at 22 °C (MOI 10:1). CFU were quantified at 1, 24, 48 and 96 hours post infection. n= 3 independent experiments. **(B)** Average colony diameter of original (left), early-adapted (middle) and late-adapted MAH colonies were measured on a Laxco SeBa Pro4B microscope at 4X magnification. Significance determined as compared to original MAH. n=20 individual colonies measured per isolate.

Figure S2. Phylogenetic tree representing 56 MAH H87 isolates. Scale bar represents 4,000 single nucleotide polymorphisms (SNPs). *M. avium* 'Chester' is a reference isolate that was originally isolated from an infected hen. *M. avium* H87 is the parent isolate published in Zhao *et al*., 2017. "Original MAH" refers to the parent isolate with *gfp.* "Early-Adapted MAH" refers to MAH recovered from *A. lenticulata* after two weeks of co-culture. "Late-Adapted MAH" refers to MAH recovered from *A. lenticulata* after 42 weeks of co-culture. "Early-Adapted MAH After 24 Hour Infection" indicates early-adapted MAH recovered from a second infection of naïve *A. lenticulata* after 24 hours of co-culture. "Late-Adapted MAH After 24 Hour Infection" refers to late-adapted MAH recovered from a second infection of naïve *A. lenticulata* after 24 hours of co-culture. The last number in group sample ID refers to the colony number picked from a culture plate.

Figure S3. 16S rDNA and mycobacteria-specific rpoB PCR of *A. lenticulata* and culture testing. (A) 16S rRNA PCR gel electrophoresis of uninfected *A. lenticulata* collected at weeks 2, 13, 42 along with a sample from a current *Acanthamoeba* culture. DNA was extracted from an early-adapted (2 week) *A. lenticulata* culture infected with *M. avium* subsp. *hominissuis* and *M. avium* subsp. *hominissuis* only DNA was also included as controls. NTC = no template control. Each sample was run in duplicate ("R1 or R2"). Eukaryotic DNA ~ 900 bp. Bacterial 16S ~ 478 bp; area highlighted by white box. **(B)** Mycobacteria-specific rpoB PCR gel electrophoresis the same samples detailed in "A." NTM rpoB ~ 831 bp. **(C)** *A. lenticulata* parent culture (before

MAH infection) lysate were spread plated on TSA plates and incubated at 22 °C or 37°C. Plates were observed each day for viable growth up to 10 days **(D)**. **(E)** The remaining lysate was inoculated into TSB and incubated at 22 °C or 37 °C. Turbidity was monitored up to 10 days. Images shown were taken immediately after vortexing the cultures for 10 seconds.

Figure S4: Adaptation to *A. lenticulata* does not affect MAH cell wall lipid or protein profiles. (A) TLC of total cell wall lipid species or glycolipids from original, early- and late-adapted MAH isolated across one year of *A. lenticulata* co-culture. Plates were run using 65:24:4 chloroform:methanol:H_2O on silica plates. Total lipids were visualized with $CuSO_4$ (lipid species) or α-naphthol (glycolipids). Samples were loaded at origin eluted towards the top (solvent front). Banding indicates polar (bottom) and nonpolar (top) lipids based on affinity to silica. **(B)** SDS-PAGE and Imperial^{TM} protein stain (Invitrogen) performed on 20 µg of total protein isolated from original, early-adapted and late-adapted MAH. Red arrows indicate regions in which protein profiles differ between MAH isolates.

Figure S5: Reduced survival of MAH in a different *Acanthamoeba* species, but no difference at 22 ˚C incubation. Original, early-adapted and late-adapted MAH were used to infect naïve cultures of **(A)** *A. lenticulata* or **(B)** *A. castellanii* incubated at 22 ºC. **(C)** $1x10^7$ CFU of original and late-adapted MAH were inoculated into PYG broth incubated at 22 ºC (left) or 37 ºC (right) for 96 hours.

TABLE S1. MAH isolate metadata.

Stage	Isolate	Assembly Length (bp)	Contigs	Annotated Genes	NCBI SRA#
**Original MAH**	GFPH87-1	5,396,826	92	5,117	TBD
	GFPH87-2	5,396,766	91	5,109	TBD
	GFPH87-3	5,395,550	98	5,105	TBD
	GFPH87-4	5,395,562	97	5,106	TBD
	GFPH87-5	5,394,492	97	5,122	TBD
	GFPH87-6	5,396,843	89	5,110	TBD
**Early-adapted MAH**	NJH87-ALMA-1-1	5,319,803	291	5,111	TBD
	NJH87-ALMA-1-10	5,329,000	196	5,116	TBD
	NJH87-ALMA-1-11	5,365,171	202	5,134	TBD
	NJH87-ALMA-1-12	5,354,094	231	5,132	TBD
	NJH87-ALMA-1-13	5,372,460	182	5,125	TBD
	NJH87-ALMA-1-14	5,381,461	159	5,134	TBD
	NJH87-ALMA-1-15	5,379,375	146	5,132	TBD
	NJH87-ALMA-1-16	5,382,613	139	5,127	TBD
	NJH87-ALMA-1-17	5,383,965	140	5,118	TBD
	NJH87-ALMA-1-18	5,380,229	153	5,132	TBD
	NJH87-ALMA-1-19	5,382,275	157	5,123	TBD
	NJH87-ALMA-1-2	5,347,212	263	5,161	TBD
	NJH87-ALMA-1-20	5,383,321	146	5,114	TBD
	NJH87-ALMA-1-3	5,352,706	231	5,136	TBD
	NJH87-ALMA-1-4	5,363,433	215	5,131	TBD
	NJH87-ALMA-1-5	5,365,288	201	5,134	TBD
	NJH87-ALMA-1-6	5,347,933	213	5,128	TBD
	NJH87-ALMA-1-7	5,354,536	227	5,133	TBD
	NJH87-ALMA-1-8	5,361,190	198	5,128	TBD
	NJH87-ALMA-1-9*	5,287,721	365	5,150	TBD
**Late-adapted MAH**	NJH87-ALMA-2-1	5,376,758	153	5,128	TBD
	NJH87-ALMA-2-10	5,382,647	126	5,121	TBD
	NJH87-ALMA-2-11*	5,342,933	137	5,087	TBD
	NJH87-ALMA-2-12	5,380,171	146	5,128	TBD
	NJH87-ALMA-2-13	5,380,135	145	5,128	TBD
	NJH87-ALMA-2-14	5,383,378	130	5,113	TBD
	NJH87-ALMA-2-15	5,392,838	111	5,113	TBD
	NJH87-ALMA-2-16	5,386,436	113	5,119	TBD
	NJH87-ALMA-2-17	5,389,142	114	5,114	TBD
	NJH87-ALMA-2-18	5,386,471	114	5,122	TBD
	NJH87-ALMA-2-19	5,390,945	109	5,118	TBD
	NJH87-ALMA-2-2	5,384,995	135	5,132	TBD
	NJH87-ALMA-2-20	5,386,952	111	5,114	TBD
	NJH87-ALMA-2-3	5,387,872	119	5,120	TBD
	NJH87-ALMA-2-4	5,387,497	119	5,117	TBD
	NJH87-ALMA-2-5	5,384,710	118	5,119	TBD
	NJH87-ALMA-2-6	5,384,536	120	5,126	TBD
	NJH87-ALMA-2-7	5,384,339	123	5,128	TBD
	NJH87-ALMA-2-8	5,383,490	125	5,123	TBD
	NJH87-ALMA-2-9	5,358,479	214	5,148	TBD
**Early-adapted After 24 Hour Infection**	E-ALMA1	5,396,319	91	5,105	TBD
	E-ALMA2*	3,855,364	1,670	4,428	TBD
	E-ALMA3	5,391,571	102	5,117	TBD
	E-ALMA4	5,389,486	108	5,122	TBD
	E-ALMA5	5,392,346	91	5,116	TBD
	E-ALMA6	5,395,883	87	5,113	TBD
**Late-adapted After 24 Hour Infection**	L-ALMA1	5,395,453	95	5,102	TBD
	L-ALMA2	5,395,646	95	5,099	TBD
	L-ALMA3	5,396,198	90	5,100	TBD
	L-ALMA4	5,396,135	91	5,102	TBD
	L-ALMA5	5,301,927	92	5,016	TBD
	L-ALMA6	5,395,914	92	5,110	TBD

Genome assembly lengths, contigs and annotated genes found in the original, early-adapted, late-adapted and 24 hour infection MAH. Asterisk (*) indicates isolates excluded from analyses due to sequencing quality control metrics (low read-coverage, high assembly contig numbers, unusual assembly length).

TABLE S2. Intergenic single nucleotide polymorphisms (SNPs) between experimental stages of MAH infection and 24 hour infection of *A. lenticulata*.

Coordinate	Original MAH Vs Early-adapted MAH	Early-adapted MAH Vs Late-adapted MAH	Early-adapted MAH Vs 24 Hr Infection	Late-adapted MAH Vs 24 Hr Infection	Mean F_{ST}
21094	0.03	0.00	0.37	0.31	0.18
21095	0.04	0.04	0.00	0.31	0.06
21096	0.04	0.04	0.00	0.31	0.06
21097	0.02	0.04	0.26	0.31	0.13
21102	0.04	0.04	0.06	0.02	0.04
21103	0.04	0.04	0.06	0.03	0.03
21104	0.04	0.04	0.06	0.03	0.03
93579	0.00	0.13	0.00	0.06	0.02
99293	1.00	0.00	1.00	0.92	0.73
163962	0.11	0.13	0.09	0.21	0.08
230433	0.26	0.07	0.38	0.90	0.40
230437	0.00	0.04	0.02	0.38	0.08
230438	0.00	0.06	0.02	0.12	0.01
230439	0.00	0.05	0.02	0.10	0.01
230440	0.00	0.04	0.02	0.57	0.13
230441	0.02	0.05	0.19	0.64	0.21
230442	0.02	0.05	0.19	0.64	0.21
230444	0.02	0.06	0.06	0.57	0.11
230445	0.02	0.02	0.19	0.53	0.17
233487	0.04	0.06	0.00	0.04	0.03
233489	0.12	0.04	0.19	0.06	0.08
233490	0.04	0.04	0.00	0.03	0.01
233491	0.04	0.06	0.26	0.02	0.04
565624	0.90	0.00	0.57	0.49	0.49
916159	0.16	0.03	0.11	0.03	0.01
963263	0.27	0.05	0.07	0.00	0.10
963264	0.27	0.05	0.37	0.00	0.17
963265	0.06	0.02	0.13	0.03	0.03
963268	0.38	0.01	0.50	0.03	0.23
963269	0.45	0.00	0.58	0.26	0.32
963270	0.30	0.06	0.16	0.17	0.17
963272	0.08	0.02	0.83	0.21	0.28
971942	0.17	0.06	0.00	0.10	0.03
1010503	0.01	0.04	0.34	0.18	0.12
1025514	0.35	0.08	0.27	0.80	0.37
1025517	0.37	0.05	0.12	0.59	0.28
1025518	0.37	0.05	0.12	0.59	0.28
1025520	0.35	0.09	0.11	0.59	0.29
1025521	0.37	0.05	0.32	0.59	0.33
1025522	0.37	0.05	0.32	0.59	0.33
1671961	0.17	0.16	0.00	0.04	0.07
1673359	0.64	0.03	0.33	0.52	0.38
1673361	0.79	0.03	0.77	0.52	0.53
1673363	0.64	0.06	0.33	0.26	0.32
1673364	0.79	0.03	0.58	0.32	0.43
1808108	0.05	0.03	0.01	0.11	0.02
2123981	0.06	0.03	0.20	0.00	0.04
2123982	0.06	0.03	0.20	0.00	0.04
2384394	0.11	0.04	0.11	0.06	0.08
2385728	0.64	0.03	0.76	0.12	0.39
2407681	0.04	0.00	0.28	0.06	0.08
3006086	0.04	0.05	0.08	0.08	0.04
3023319	0.03	0.04	0.08	0.00	0.04
3023328	0.22	0.04	0.01	0.12	0.08
3152940	0.08	0.06	0.07	0.08	0.04
3152941	0.08	0.06	0.07	0.03	0.02
3152942	0.08	0.06	0.07	0.08	0.04
3152943	0.08	0.06	0.07	0.08	0.04
3152947	0.08	0.06	0.07	0.08	0.04
3152948	0.08	0.06	0.07	0.08	0.04
3571894	0.35	0.01	0.06	0.11	0.07
3571895	0.35	0.01	0.06	0.11	0.07
3571896	0.23	0.04	0.07	0.04	0.02
3571898	0.12	0.01	0.01	0.04	0.02

3571899	0.04	0.04	0.07	0.04	0.03
3572877	0.00	0.00	0.00	0.08	0.02
3715080	0.00	0.00	0.00	0.03	0.01
4236654	0.02	0.08	0.07	0.08	0.01
4236656	0.02	0.13	0.07	0.12	0.01
4236660	0.10	0.08	0.13	0.06	0.05
4236661	0.10	0.08	0.13	0.06	0.05
4421904	0.79	0.00	0.78	0.92	0.63
4422260	0.03	0.07	0.05	0.21	0.05
4461897	0.08	0.01	0.10	0.03	0.06
4461898	0.03	0.01	0.10	0.03	0.04
4552312	0.06	0.03	0.04	0.03	0.01
4552326	0.00	0.03	0.00	0.06	0.01
4552352	0.21	0.03	0.60	0.52	0.32
4552355	0.21	0.03	0.60	0.52	0.32
4562156	0.90	0.00	1.00	0.80	0.68
4562157	0.07	0.01	0.08	0.18	0.00
4591436	0.05	0.10	0.01	0.03	0.03
4591439	0.10	0.04	0.01	0.01	0.04
4712269	0.00	0.00	0.00	0.08	0.02
4712270	0.00	0.00	0.00	0.08	0.02
5385581	0.48	0.05	0.32	0.42	0.29

For all SNPs located between genes in the MAH genome, grey shading indicates the coordinates of each SNP that, on average, indicate high differentiation (Mean $F_{ST} \geq 0.20$) between different experimental stages.
